# Replantation of an Avulsed Tooth: A Case Report

**DOI:** 10.7759/cureus.39198

**Published:** 2023-05-18

**Authors:** Nishad Kadulkar, Rubi Kataki, Adrija Deka, Salouno Thonai

**Affiliations:** 1 Conservative Dentistry and Endodontics, Regional Dental College, Guwahati, IND

**Keywords:** traumatic dental injury, avulsion, replantation, storage media, extra-oral time

## Abstract

Avulsion is a rare and serious traumatic dental injury that needs immediate and complex management. This case report highlights the successful management of an avulsed maxillary central incisor by replantation after a lapse of 120 minutes of being in an extra-oral environment and preserved in milk. A female patient aged 17 years presented with a traumatic dental injury sustained to the anterior maxillary region due to an accidental fall. Clinical examination revealed an avulsed tooth 21 that was replanted according to the International Association of Dental Traumatology (IADT) guidelines and stabilised in place with splinting. Conventional root canal therapy was initiated one week post-replantation. The root canal treatment was completed two weeks post-replantation followed by removal of the splint. Follow up done at regular intervals of one, three, six, and 12 months showed a lack of clinical signs and symptoms and no resorption on radiographic interpretation.

## Introduction

Dento-alveolar trauma results from a sudden blow to the teeth and its associated structures which may lead to varied injuries including complete loss of tooth from its socket. Traumatic injuries involving dentition are seen frequently in the age group of 7-11 years. The majority of these injuries result from accidental falls followed by various sports activities, road traffic accidents, and physical assault [1]. Avulsion is one such serious traumatic dental injury that corresponds to the complete displacement of the tooth out of the alveolar bone socket. Avulsion of permanent teeth is relatively rare and varies from 0.5% to 16% in all traumatic dental injuries. Maxillary central incisors are the most commonly involved teeth due to their prominent position in the arch [2,3].

The primary line of treatment for the management of an avulsion injury is replantation. However, in certain circumstances (high caries index, periodontitis, severe cardiac or immunosuppressive conditions, apprehensive patients), this option is contraindicated. Albeit a few replanted cases have shown less long-term survival rate, not replanting a tooth is an irreversible decision. Thus, every attempt must be made to save it [4]. Also, a retrospective study in children has confirmed that the replantation procedure has a long-term survival rate (79.3%) in accordance with the treatment guidelines prescribed by the International Association of Dental Traumatology (IADT) [5]. General health of the patient, maturity of the root apex, storage medium, and extra-oral time are the factors that affect the successful outcome of the replantation procedure [6-8].

The periodontal ligament (PDL) cells left on the root surface of an avulsed tooth after trauma must be prevented from dehydration to maintain their function and viability [2]. Storage media and extra-oral time are the two most critical factors that affect the condition of the PDL cells. Milk, Hank's balanced salt solution (HBSS), Viaspan, Propolis, saliva, or saline are the storage media in which the avulsed tooth can be stored till the time it is replanted [1,9]. Amongst these, milk being easily available and having a suitable pH with appropriate growth factors, nutrients, and osmolarity, is the most extensively used and recommended storage media [10]. The survival of PDL cells is adversely affected by an extended extra-oral dry time that is longer than one hour, with root resorption (inflammatory/replacement) being a potential complication [3,4]. This case report highlights the comprehensive procedures executed in the successful replantation of an avulsed maxillary central incisor despite an extended extra-oral duration of 120 minutes.

## Case presentation

**Figure 1:**
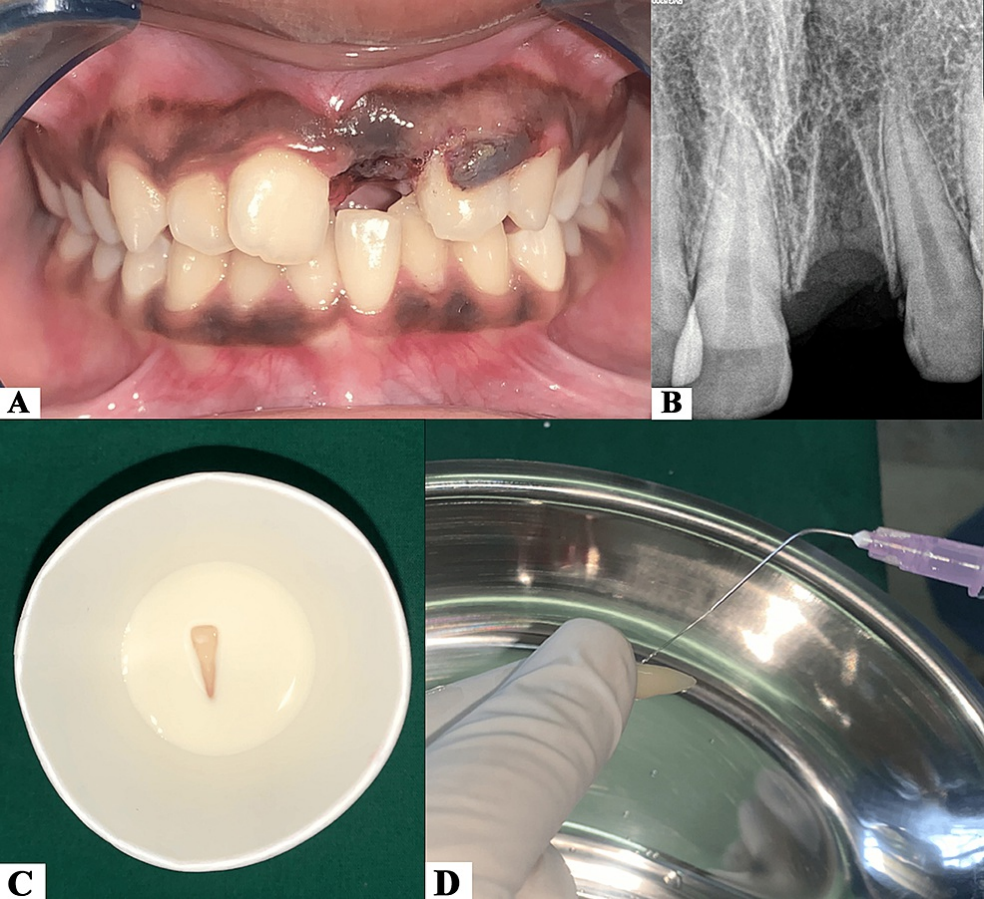
(A) Pre-operative clinical image. (B) Pre-operative radiograph showing empty alveolar socket in 21 region. (C) Avulsed tooth stored in milk. (D) Root surface being rinsed with normal saline.

A 17-year-old female patient presented with an alleged history of fall and injuries sustained to the anterior maxillary region. The patient had no relevant past medical history and was alert as well as responsive during the process of examination. Extra-oral examination revealed mild abrasion and swollen upper lip. On intra-oral examination, the left maxillary central incisor (21) was missing and the marginal gingiva in the associated area was lacerated (Figure 1A). On inspection and palpation of the anterior maxillary segment, the dento-alveolar fracture was ruled out. Radiovisiography (RVG) revealed an empty alveolar socket with an intact lamina dura in the 21 region; there was no other injury or fracture of the adjacent teeth and associated alveolar structures (Figure 1B). The case was diagnosed as an Ellis class V fracture with 21 [11]. The patient preserved the avulsed tooth in milk and reported to the Department of Conservative Dentistry and Endodontics at the dental college approximately two hours after the incidence of trauma (Figure 1C). The patient was informed about the plausible complications (inflammatory resorption, replacement resorption/ankylosis, tooth discolouration) involved with replanting an avulsed tooth that had endured an extra-oral time of approximately 120 minutes. After obtaining informed consent, it was decided to reposition and replant the avulsed tooth. The avulsed tooth had an intact crown and a well-formed root with a closed apex. After taking it out from the milk, the avulsed tooth was held by the crown, taking care that the root surface was not touched. The root surface was then gently rinsed with normal saline in order to remove any debris that had adhered to it (Figure 1D).

**Figure 2:**
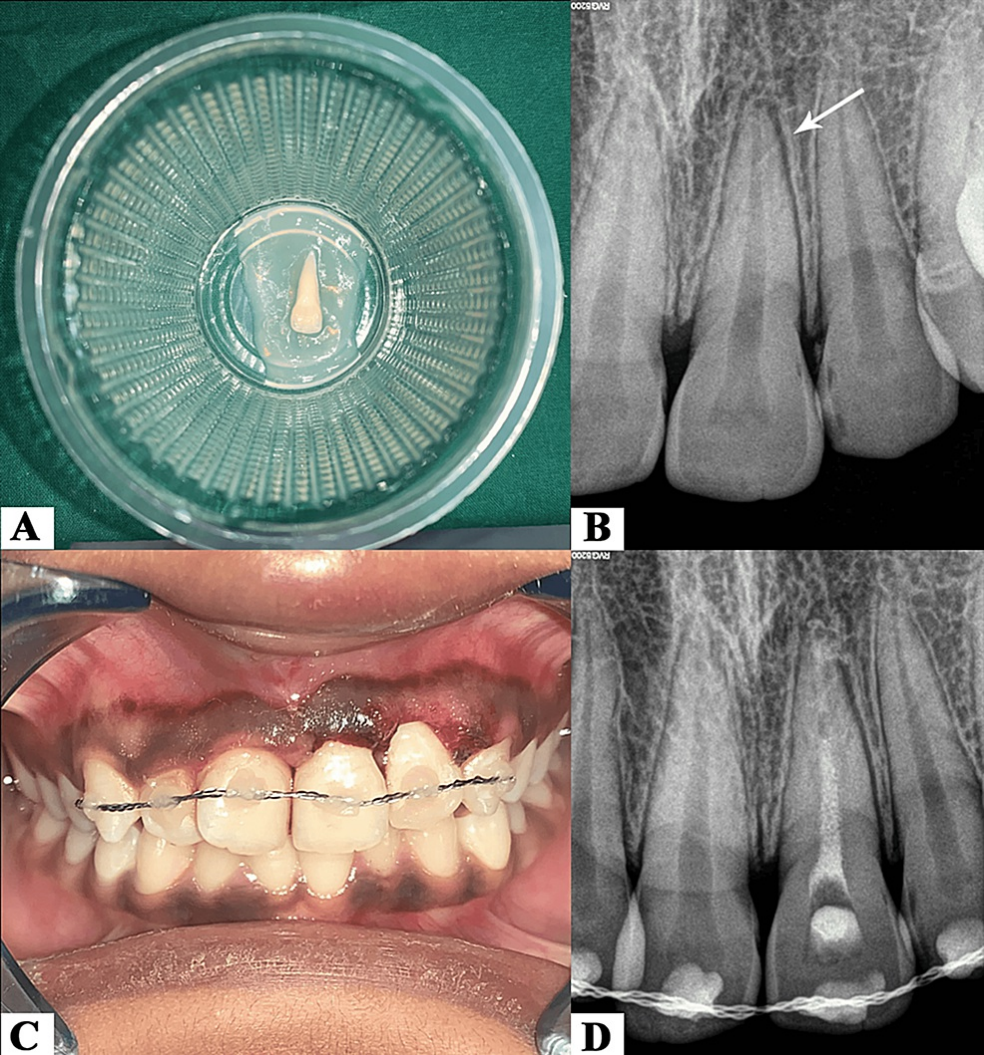
(A) Root surface treatment with 1.23% acidulated phosphate fluoride (APF) gel. (B) Radiographic verification for correct positioning of 21 in its alveolar socket. (C) Replanted tooth stabilised in place with splinting. (D) Intracanal medicament placed in 21.

The root surface of the avulsed tooth was treated with 1.23% acidulated phosphate fluoride (APF) gel for a duration of 20 minutes (Figure 2A). Local anaesthesia (2% lignocaine without vasoconstrictor) was administered with labial and palatal infiltration in the involved area. The alveolar socket was gently rinsed with normal saline. The avulsed tooth was then repositioned in the socket with slight digital pressure and the correct positioning was verified with RVG (Figure 2B). The repositioned tooth had an acceptable occlusion and thus, occlusal adjustment was not required. After radiographic verification, the replanted tooth was stabilised in its socket with splinting that comprised of an orthodontic wire (0.4 mm stainless steel wire) secured with light-cure flowable composite resin (Brilliant Flow, Coltene, Altstatten, Switzerland). The labial surfaces of the maxillary anterior teeth were spot etched at the middle third level of the crown with 37% phosphoric acid for 20 seconds which was rinsed and air dried. Bonding agent (ScotchBond Universal adhesive, 3M ESPE, Bayern, Germany) was then applied and light cured for 20 seconds. Flowable composite was placed at the respective spots and then the orthodontic wire was secured in place after light curing each tooth for 20 seconds (Figure 2C). Oral analgesics and antibiotics (amoxicillin 500 mg + clavulanic acid 125 mg twice daily and metronidazole 400 mg thrice daily) were prescribed for five days. The patient was also recommended to seek an anti-tetanus booster dose. She was advised to maintain a soft diet for two weeks along with the use of a soft-bristled toothbrush and 0.12% chlorhexidine mouth rinse for oral hygiene maintenance. The patient was recalled after a week and conventional root canal therapy was initiated in the replanted tooth. After administration of local anaesthesia, an access cavity was prepared followed by extirpation of the pulp and working length determination with #15 K file (Dentsply Maillefer, Ballaigues, Switzerland). The root canal space was shaped and prepared with rotary instrumentation till size F2 of the ProTaper Gold rotary file system (Dentsply Maillefer, Ballaigues, Switzerland). After each instrument change, the root canal space was irrigated with 2.5 ml of 3% sodium hypochlorite (NaOCl) solution followed by a final rinse with 17% ethylenediamine tetra-acetic acid (EDTA) solution for a minute. After rinsing with saline, the canal was dried with absorbent paper points. Calcium hydroxide intracanal medicament was placed in the root canal space with a lentulo spiral and the access cavity sealed with a temporary filling (Tempodent, TechnoDent, Maharashtra, India) (Figure 2D).

**Figure 3:**
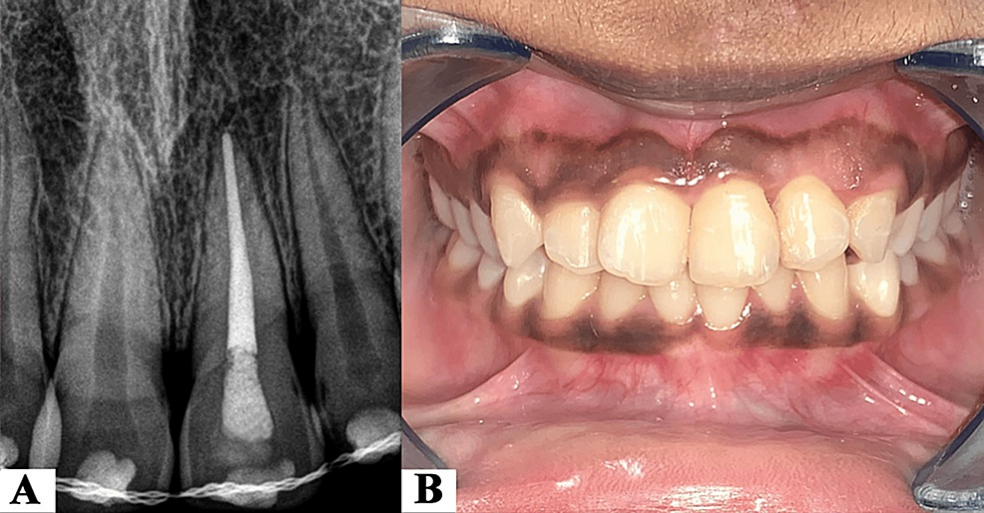
(A) Radiograph showing completed root canal therapy with 21. (B) Intra-oral clinical image after completion of root canal therapy and removal of splint.

Two weeks after replantation, the intracanal medicament was removed. The root canal space of the replanted tooth was obturated with gutta-percha and epoxy-resin sealer (Dentsply Maillefer, Ballaigues, Switzerland) by the cold lateral compaction technique. The access cavity was then sealed with a light-cure composite resin restoration (Filtek P60, 3M ESPE, Bayern, Germany) (Figure 3A). The splint was removed and a vitality test was performed on the adjacent teeth that elicited a positive response to electric pulp testing (EPT) (Figure 3B). It was also noted that the mobility of the replanted tooth had reduced to grade I.

**Figure 4:**
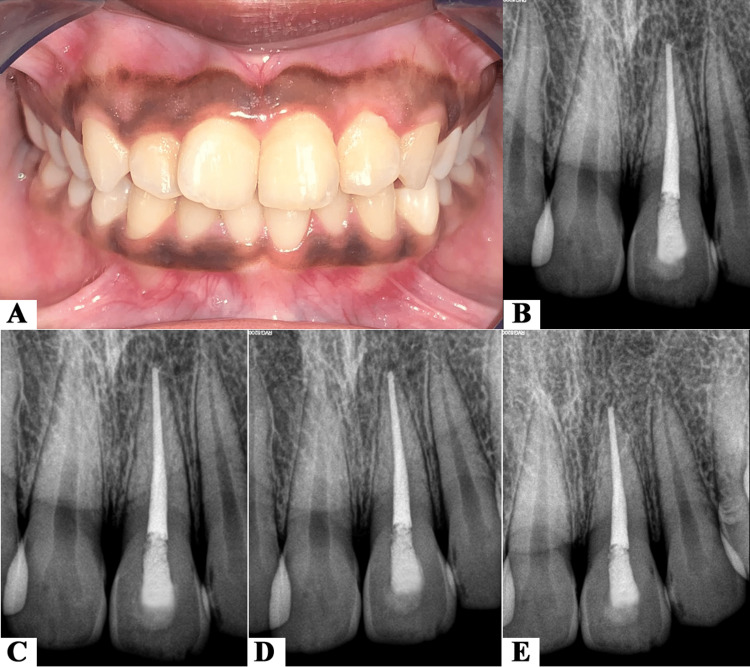
(A, B) Clinical and radiographic images at one month follow-up. (C) Radiograph at three months follow-up. (D) Radiograph at six months follow-up. (E) Radiograph at 12 months follow-up.

At one-month follow-up visit, clinical examination showed normal physiologic mobility and normal probing sulcular depth with the replanted tooth (Figure 4A). Radiographic examination revealed normal periapex with no associated periapical radiolucency, an intact lamina dura with uniform PDL space around 21, and no signs of resorption (Figure 4B). These findings were consistent at three months, six months, and 12 months follow-up visits (Figures 4C-4E).

## Discussion

The optimal treatment option following an avulsion injury is to replant the avulsed tooth at the earliest. However, this is not feasible in all instances. This case report focuses on the replantation of an avulsed maxillary central incisor after an extra-oral time of nearly two hours.

Appropriate emergency management, significant treatment plans, and prompt action influence good prognosis in the management of an avulsion injury [4]. The factors affecting the successful outcome of replantation are the patient’s general health, maturity of the root, storage media, and extra-oral time; with the last two factors being the most critical [6]. Amongst the various storage media available for preserving an avulsed tooth, milk being easily available and having a suitable pH with appropriate growth factors, nutrients, and osmolarity, is the most extensively used and recommended one. Also, milk being a gland secretion contains epithelial growth factor (EGF) that stimulates the proliferation and regeneration of epithelial cell rests of Malassez [12].

The extra-oral period has a direct co-relation with the survival of PDL cells. Studies have found that tooth replanted within five minutes had the best prognosis [13]. Whereas, delayed replantation results in PDL cell death when the extra-oral duration exceeds one hour [4,14]. Thus, it is crucial to preserve the avulsed tooth in an ideal storage medium with replantation being initiated at the earliest. In the current case, the patient had carried the avulsed tooth in milk and reached the department nearly two hours after the incidence of trauma. Delayed replantation causes necrosis of the PDL cells resulting in complications such as inflammatory/replacement root resorption [3,4]. These complications can be avoided by root surface treatment of the avulsed tooth with fluoride [15]. The exact mechanism is not known but it is hypothesized that fluoride converts hydroxyapatite into fluorapatite by direct action on dentin, cementum, and bone. It is also believed that fluoride specifically inhibits the activity of clastic cells [16]. In our case, the root surface of the avulsed tooth was treated with 1.23% APF gel for 20 minutes in order to prevent root resorption post-replantation. Studies have shown that the chances of complications were higher in a tooth with an immature apex than in a mature tooth [17]. In the current case, this factor was favourable as the avulsed tooth had a closed apex.

The rationale behind splinting the replanted tooth is to stabilise it in its appropriate position. In the case discussed above, splinting was done for two weeks with 0.4 mm stainless steel orthodontic wire bonded to the maxillary anterior teeth with a light cure flowable composite resin. This was in accordance with the guidelines prescribed by IADT [4]. The ideal duration of splinting a replanted avulsed tooth is believed to be two weeks because studies have shown that more than 60% of the mechanical properties of the injured PDL return within two weeks following injury [18]. However, an additional week of splinting would be necessary in case of excessive trauma or if it is unable to maintain the avulsed tooth in its correct position [4].

As per the IADT guidelines, root canal therapy must be initiated within two weeks post-replantation. The replanted tooth needs endodontic therapy because the necrotic pulp and its toxins may gain access to the periodontal ligament through various portals of exit, thus contributing to the process of resorption [15]. In the past, it was advised to perform root canal therapy extra-orally before replantation. However, the current guidelines recommend root canal therapy be performed intra-orally. This minimizes the extra-oral time and associated risk factors [4]. In our case, endodontic treatment was initiated one week after replantation followed by placement of calcium hydroxide intracanal medicament for one week. Calcium hydroxide has antimicrobial effects, inhibits bacterial enzymes, activates tissue enzymes such as alkaline phosphatase, and stimulates mineralisation; thus, helping in thorough disinfection and reducing the chances of replantation-associated root resorption [19]. Although the current guidelines recommend placing calcium hydroxide for a longer duration of four weeks, it has been shown to have similar efficacy when placed for a shorter duration in the absence of pathology [20].

A replanted tooth must be followed up at regular intervals till 12 months and thereafter annually for a minimum period of five years, with clinical and radiographic examinations at every follow-up visit to rule out any associated complications [4]. Educating the patient regarding emergency management following avulsion and various storage media is essential for the successful management of an avulsed tooth.

## Conclusions

Replantation is the treatment of choice following avulsion. It not only satisfies the patient’s functional and aesthetic concerns but also helps to maintain the surrounding bone for prosthetic rehabilitation, in case of replantation failure. Despite an extended extra-oral time, replantation of an avulsed tooth can have a favourable outcome if all the recommended guidelines and protocols are followed.
